# Variability of E2 protein-coding sequences of bovine viral diarrhea virus in Polish cattle

**DOI:** 10.1007/s11262-020-01756-2

**Published:** 2020-04-16

**Authors:** Paweł Mirosław, Mirosław P. Polak

**Affiliations:** Department of Virology, National Veterinary Research Institute, Al. Partyzantów 57, 24-100 Puławy, Poland

**Keywords:** Bovine viral diarrhea virus, E2 glycoprotein, Pestivirus, Polymorphism, Sequencing

## Abstract

Bovine viral diarrhea virus (BVDV) belongs to the *Pestivirus* genus of the *Flaviviridae* family and has worldwide distribution, being one of the main causes of economic losses in cattle raising. The genome of pestiviruses is a single strand of positive-sense RNA with a length of 12.3 kb, which encodes one open reading frame flanked by untranslated regions. E2 glycoprotein is required for binding to cell-surface receptors and it also contains major antigenic determinants. The nucleotide sequence coding E2 is the most variable part of the viral genome. The heterogeneity that exists among circulating strains causes problems in the development of effective vaccines and reliable diagnostics. In this study, and for the first time analysis was made of the E2 glycoprotein coding sequences of 14 Polish BVDV-1 strains which belong to four subtypes: 1b (*n* = 7), 1f (*n* = 3), 1s (*n* = 3), and 1r (*n* = 1). These sequences showed evidence of strong purifying (negative) selection. However, we also identified positively selected sites. The availability of E2 sequences of Polish BVDV strains for reference, knowledge gained through epitope prediction attempts, and information on protein glycosylation sites can afford a better understanding of host–pathogen interactions.

Bovine viral diarrhea virus (BVDV) is one of the most widespread viruses in cattle breeding and causes large economic losses [1]. In Europe, the prevalence of antibody-positivity in animals in countries without systemic BVD control is between 60 and 85% [2]. BVDV can induce many different clinical symptoms in infected animals. Usually, these symptoms are mild, although hemorrhagic syndrome and mucosal disease can manifest in the rare severe cases [3]. BVDV species 1 (BVDV-1, Pestivirus A) and 2 (BVDV-2, Pestivirus B) belong to the *Pestivirus* genus of the *Flaviviridae* family together with classical swine fever virus (CSFV, Pestivirus C) and border disease virus (BDV, Pestivirus D) [4]. The genetic material of pestiviruses is a single-stranded positive-sense RNA with a length of 12.3 kb, which encodes one open reading frame flanked by untranslated regions from both ends.

A virion has a diameter of 40–60 nm and it is surrounded by a lipid membrane. The lipid membrane contains three glycosylated envelope proteins: E^rns^, E1, and E2 [5]. The two envelope glycoproteins E1 and E2 recognize host cells by binding to cell-surface receptors CD46 and LDL-R [6] and are required for membrane fusion and cell entry [7]. E1 is assumed to function as a membrane anchor for E2 [8], which also contains major antigenic determinants [9, 10]. The BVDV genome has a high mutation rate and the E2 glycoprotein coding fragment is the most variable part of its genome. The heterogeneity that exists among circulating strains causes problems in the development of effective vaccines and sensitive diagnostics.

The present study is the first to provide sequence information for the E2 glycoprotein of Polish BVDV strains belonging to the most often isolated subtypes in Poland. We focused on determining which E2 glycoprotein regions are subject to positive selection and the detection of protein glycosylation sites. This data may be a key indicator of the nature of the host–virus interaction. In this study, we used BVDV-positive serum samples from previous detection and genotyping studies of clinical suspects, herds with virus eradication underway and herds vaccinated with killed BVDV-1a vaccine [11].

Total RNA was extracted using TRI Reagent (Sigma-Aldrich, USA) from 500 µL of serum following the manufacturer’s instructions. Reaction mixes for standard RT-PCR were prepared as described previously [11]. A mix of four primer pairs specific to the E2-encoding fragment [12] and specific to regions flanking the E2 encoding sequence [13–15] was used. A list of primer sequences is presented in Table 1. We obtained positive RT-PCR results for 16 out of 30 samples in the form of a band on agarose gel with a size of about 1019–1200 nucleotides. However, for further studies, it was only possible to use 14 viral sequences, as only this many were of good quality, and these were submitted to GenBank with the accession numbers MK675059–MK675072. The list of strains for which sequences were obtained can be found in Table 2, where the geographical origin of the samples is given. The analyzed sequences were assigned to four groups on the phylogenetic tree (Fig. 1), to the same subtypes as in the previous study within 5′UTR: 1b (*n* = 7), 1f (*n* = 3), 1s (*n* = 3), and 1r (*n* = 1) [11]. Subtype 1b is currently the most often isolated subtype of BVDV in Poland. Almost a quarter of all isolated viruses belonged to the 1f subtype. The remaining two subtypes, 1r and 1s, were identified recently and are rare.

**Table 1:** List of primers used in the study

Primer	Sequence 5′–3′	Reference
F1	AGCACTGAGGGGACAACTAAT	Pecora et al. [15]
R1	GCCTATCATGACTATCTCTTCAGT	
R2	TTCAGTATTCACTCCAGCACC	
738F	TRTGGCTGCTACTAGTAACNGGGGCACAAGG	van Rijn et al. [13]
810F	TGGCTACTACTAGTAACAGGGGTACAAGG	
BVIIR	GTRAGCAAGTTGCCYATCATYAC	Tijssen et al. [14]
2274F	TGGTGGCCTTATGAGAC	Nagai et al. [12]
3434R	AGGTCAAACCAARTATTG

**Table 2:** Field strains for which the sequences of the E2 region were obtained and reference strains retrieved from GenBank for comparative analysis

Field strains						
Species	Herd	Strain	Subtype	Region	Vaccination	Accession number
BVDV-1	1	164-DM/15	1f	Lublin Voivodeship	No	MK675059
BVDV-1	1	165-DM/15	1f		No	MK675060
BVDV-1	2	166-KY/15	1s	Kuyavian-Pomeranian Voivodeship	No	MK675061
BVDV-1	2	167-KY/15	1s		No	MK675062
BVDV-1	3	176-KR/15	1s		No	MK675063
BVDV-1	4	177-EP/16	1b	Lublin Voivodeship	No	MK675064
BVDV-1	5	179-WD/17	1f	Lublin Voivodeship	No	MK675065
BVDV-1	6	183-SY/17	1r	Świętokrzyskie Voivodeship	No	MK675066
BVDV-1	7	186-km/17	1b	Wielkopolska Voivodeship	No	MK675067
BVDV-1	8	187-AN/17	1b	Wielkopolska Voivodeship	Yes	MK675068
BVDV-1	9	194-TC/17	1b	Wielkopolska Voivodeship	Yes	MK675069
BVDV-1	10	200-BA/17	1b	Wielkopolska Voivodeship	No	MK675070
BVDV-1	11	207-LK/18	1b	Wielkopolska Voivodeship	No	MK675071

Reference strains						
Species	Strain		Subtype	Accession number		
BVDV-1	NADL		1a	M31182.1		
BVDV-1	Singer		1a	DQ088995.2		
BVDV-1	C86		1a	Y19123.1		
BVDV-1	Oregon C24V		1a	AF091605.1		
BVDV-1	VEDEVAC		1b	AJ585412.1		
BVDV-1	Osloss		1b	M96687.1		
BVDV-1	KE9		1b	EF101530.1		
BVDV-1	New York-1 (NY-1)		1b	AY027671.1		
BVDV-1	XZ02		1b	MF278652.1		
BVDV-1	Braidwood		1c	AF255049.1		
BVDV-1	Bega		1c	AF049221.2		
BVDV-1	519		1c	AF144610.1		
BVDV-1	10JJ-SKR		1d	KC757383.1		
BVDV-1	BJ1308		1d	KT951841.1		
BVDV-1	SLO/2407/2006		1e	KX577637.1		
BVDV-1	Carlito		1e	KP313732.1		
BVDV-1	SLO/1170/2000		1f	KX987157.1		
BVDV-1	UM/103/04		1g	LT797813.1		
BVDV-1	UM/126/07		1h	LT631725.1		
BVDV-1	ACM/BR/2016		1i	KX857724.1		
BVDV-1	KS86-1ncp		1j	AB078950.1		
BVDV-1	KS86-1cp		1j	AB078952.1		
BVDV-1	SuwaCP		1k	KC853441.1		
BVDV-1	SuwaNcP		1k	KC853440.1		
BVDV-1	ZM-95		1m	AF526381.3		
BVDV-1	SD-15		1m	KR866116.1		
BVDV-1	Shitara/02/06		1n	LC089876.1		
BVDV-1	IS26/01ncp		1o	LC089875.1		
BVDV-1	TJ41		1o	KF048848.1		
BVDV-1	LEI01		1p	KF048849.1		
BVDV-1	TJ142		1p	KF048850.1		
BVDV-1	camel-6		1q	KC695810.1		
BVDV-1	GS-3		1q	KC695811.1		
BVDV-1	VE/245/12		1r	LT837585.1		
BVDV-1	Mousedeer		1s	AY162456.1		
BVDV-2	New York-93 (NY-93)		2a	KR093034.1		
BVDV-2	793		2a	HQ174302.2

**Fig. 1:**
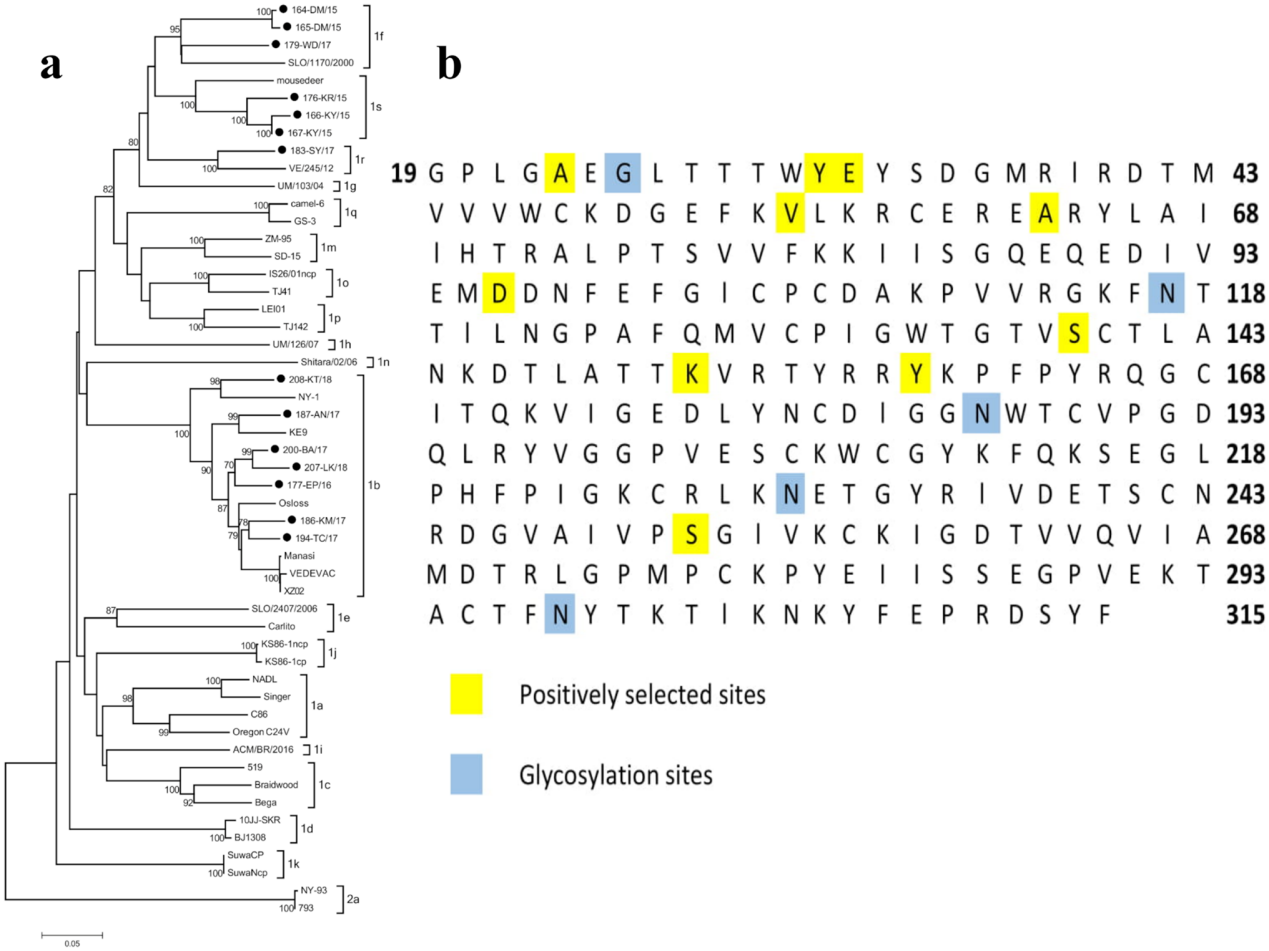
**a** Phylogenetic tree based on a 636 nt fragment of the E2 gene. Black dots indicate analyzed strains. **b** Consensus sequence of tested strains with positive selection sites and glycosylation sites

We were able to amplify the E2 region from a much smaller number of strains than was possible for the 5′ untranslated region in a previous study [11]. Due to the high variability, the E2 region is not suitable as a target for diagnostic purposes, but it can provide useful information when results from the 5′UTR are unclear. The analyzed fragment of the E2 region is longer than 5′UTR, it is less conserved, and the constructed phylogenetic tree within this region has higher bootstrap values.

The percentage of sequence identity of our strains was calculated based on a 892-nucleotide fragment (nucleotide positions 55–946 and amino acid positions 19–315 of the C86 vaccine strain in the E2 gene). The fragment analyzed is shorter than the full-length E2 sequence due to unsatisfactory sequence quality at its ends. The identity between various strains ranged from 70.4 to 98.5%, with the smallest differences occurring between subtypes 1f and 1s (79.5–81.75% identity), and the largest ones between 1b and 1f (70.4–73% identity). Differences among strains within subtypes 1b, 1s, and 1f were 84.7–96%, 94.2–98.5%, and 83.9–98.5%, respectively. The analyzed region of E2 displayed evolutionary distances of 1.5–16.1% at the subtype level and 18.3–29.6% among subtypes. Similar evolutionary distances to the Polish strains were also found among Chinese strains [16]. The E2 sequences from this study displayed 437/892 (48.9%) variable sites at the nucleic acid level: 83 singleton variable sites and 354 parsimony informative sites. The total number of mutations was 597. We identified 43.77% variable sites at amino acid level, which presented up to six amino acid variants, and these six variants occurred in positions 57, 159, and 198. Five variants appeared in 13 different positions.

We investigated the nature of selection pressure acting on the envelope glycoprotein E2 gene by the estimation of synonymous/nonsynonymous (dS/dN) mutation rates (*ω*) using DNASP6.11 [17]. The E2 glycoprotein coding region showed evidence of strong purifying selection throughout the sequence (*ω* = 0.143), i.e., stabilizing it through the removal of deleterious genetic polymorphisms that arise through random mutations. Positive selection is a type of natural selection in which a specific phenotype is preferred to other phenotypes. We have identified several locations in the genome that are characterized by positive selection (Fig. 1): 68–70 (dN/dS ratio 1.893), 91–96 (1.087), 163–165 (2.212), 170–173 (1.767), 187–189 (1.232), 286–288 (4.818), 415–417 (1.552), 454–456 (3.339), 475–477 (2.972), and 754–756 (3.301).

E2 glycoprotein, which is the most immunodominant viral protein of pestiviruses, contains major type-specific epitopes recognized by specific antibodies. This protein is presented by antigen-presenting cells and was identified as a target for cytotoxic T-cells [18]. Positive selection might contribute to the avoidance of T-cell recognition of the E2 glycoprotein. For BVDV-1, essential amino acid positions for neutralization by monoclonal antibodies (mAbs) have been mapped in the N-terminal part of E2 [19]. Two antigenic regions of the E2 glycoprotein were mapped between amino acid positions 1–70 and 70–77 [20]. In this study, we identified five sites showing positive selection in the 1–70 fragment. Other studies showed that single nucleotide mutation causing the change of one of the four amino acids in the 71–74 region can defeat neutralization by a single mAb [20]. Changes at position 72 observed in Singer BVDV-1a strain mutants affected the binding and neutralization of mAbs 157 and 348 [20]. Leucine at position 74 was also important for the binding of the WB166 antibody [21]. The Singer strain showing a different change, at position 32, managed to evade the antibody 157. Similarly, the Hastings strain of BVDV-1 also managed to escape this antibody through a change in the same position [20]. We identified mild positive selection for this position in Polish strains of BVDV-1.

The BVDV-1-specific epitope X1 recognized by mAb 921–6 [21] (60–90 in the E2 glycoprotein) was mapped in the C-terminal portion of the A domain region. In this region, we found one area showing positive selection: amino acid 63.

An immunodominant region at amino acid position 121–150, corresponding to the linear neutralizing epitope of the E2 protein of CSFV, was recognized by polyclonal antibodies [22]. There were differences in nine positions in our strains, particularly in the C-terminal part. We also observed a positive selection in this region, specifically for position 139.

Amino acids 96 and 152 showed the strongest positive selection of the strains from this study. However, identifying the importance of this selection requires further research.

The sequence coding for the receptor binding domain, which usually mediates binding of the virion to the host cell surface, is located at amino acid position 141–170 of E2 in CSFV, indicating that binding of the receptor CD46 might occur through this region [23]. It corresponds to the polymorphic 142–172 segment in the BVDV genome. We did not detect positive selection in the area of the receptor binding site, but high variability within a protein having a cell receptor affinity may contribute to a change in tropism. This would be caused by random mutations enabling the crossing of species barriers and possible infection of a wider range of hosts within even-toed ungulates (*Artiodactyla*).

Four glycosylation sites in the E2 glycoprotein predicted by NetNGlyc 1.0 have been identified in positions N117, N186, N230, and N298. One extra glycosylation site in position N25 (Fig. 1) was found in the 164-DM/15 and 165-DM/15 strains (subtypes 1f) detected in samples from the same herd (Table 2). N-linked glycosylation is crucial in protein functions, such as entry into host cells, protein antigenic properties, proteolytic processing, and protein trafficking [24].

Strains 187-AN/17 and 194-TC/17 were isolated from vaccinated animals (Table 2). The vaccine was based on the C86 strain (GenBank accession number Y19123) belonging to subtype 1a. The percentage of nucleotide sequence identity of the tested field strains and the C86 strain sequence taken from the GenBank database was 72.6–73.2% and at the protein level it was 75.4–75.7%.

BVDV vaccines are mostly based on single subtypes including BVDV-1a, BVDV-1b, or BVDV-2a. Our and other investigators’ results indicate that current vaccines based on a limited number of subtypes do not provide effective protection against other subtypes of BVD virus [25]. Humoral immune response directed against E2 is considered the main line of defense against the BVD virus circulating in the field [19]. Sequence identity between our strains and the vaccine strain C86 in the 19–90 amino acid position where many antibody-recognizable epitopes was only 55.5–63.8%. In this fragment sequence similarity percentage between our strains and the C86 vaccine strain is even lower than in the entire E2 sequence studied by us.

In summary, the present study provides information for the first time of sequences of the E2 glycoprotein of Polish BVDV strains belonging to the most often isolated subtypes in Poland. We showed that E2 glycoprotein with its high genetic variability contains fragments that are positively selected. Some of these fragments may be epitopes. We believe that the strain used in vaccine production should show greater similarity to strains circulating in a given region, and that particular emphasis should fall on similarity at sites encoding immunogenic epitopes present mainly in the E2 glycoprotein. In contrast to the most frequently studied 5′UTR and *N*^pro^ region, the positively selected BVDV sites require further analysis to establish if amino acid substitutions can lead to changes of host tropism, evasion of epitope-specific CD8 T-cell response, or avoidance of antibody recognition. Understanding the importance of amino acid changes at sites of positive selection can be helpful in studying the virulence of strains and predicting future responses to vaccine strains of BVDV.
